# Spatial dynamics of tertiary lymphoid aggregates in head and neck cancer: insights into immunotherapy response

**DOI:** 10.1186/s12967-024-05409-y

**Published:** 2024-07-24

**Authors:** Habib Sadeghirad, James Monkman, Chin Wee Tan, Ning Liu, Joseph Yunis, Meg L. Donovan, Afshin Moradi, Niyati Jhaveri, Chris Perry, Mark N. Adams, Ken O’Byrne, Majid E. Warkiani, Rahul Ladwa, Brett G.M. Hughes, Arutha Kulasinghe

**Affiliations:** 1 Frazer Institute, Faculty of Medicine, Translational Research Institute, The University of Queensland, 37 Kent Street, Woolloongabba, QLD 4102 Australia; 2 Bioinformatics Division, The Walter and Eliza Hall Institute of Medical Research, Parkville, Melbourne, VIC 3052 Australia; 3 Department of Medical Biology, Faculty of Medicine, Dentistry and Health Sciences, University of Melbourne, Parkville, VIC 3010 Australia; 4 South Australian immunoGENomics Cancer Institute, The University of Adelaide, SA, Australia; 5 Ian Frazer Centre for Children’s Immunotherapy Research, Children’s Health Research Centre, Faculty of Medicine, The University of Queensland, South Brisbane, QLD, Australia; 6 Queensland Spatial Biology Centre, Wesley Research Institute, The Wesley Hospital, Auchenflower, QLD Australia; 7 Discovery Applications, Akoya Biosciences, The Spatial Biology Company, Marlborough, MA USA; 8 The Princess Alexandra Hospital, Woolloongabba, QLD Australia; 9 Centre for Genomics and Personalised Health, School of Biomedical Sciences, Faculty of Health, Queensland University of Technology, Brisbane, QLD Australia; 10 University of Technology Sydney, NSW, Australia; 11 The Royal Brisbane and Women’s Hospital, Herston, QLD Australia; 12 School of Medicine, University of Queensland, Brisbane, QLD Australia

## Abstract

**Background:**

Recurrent/metastatic head and neck squamous cell carcinoma (R/M HNSCC) generally has a poor prognosis for patients with limited treatment options. While incorporating immune checkpoint inhibitors (ICIs) has now become the standard of care, the efficacy is variable, with only a subset of patients responding. The complexity of the tumor microenvironment (TME) and the role of tertiary lymphoid structures (TLS) have emerged as critical determinants for immunotherapeutic response.

**Methods:**

In this study, we analyzed two independently collected R/M HNSCC patient tissue cohorts to better understand the role of TLS in response to ICIs. Utilizing a multi-omics approach, we first performed targeted proteomic profiling using the Nanostring GeoMx Digital Spatial Profiler to quantify immune-related protein expression with spatial resolution. This was further characterized by spatially resolved whole transcriptome profiling of TLSs and germinal centers (GCs). Deeper single-cell resolved proteomic profiling of the TLSs was performed using the Akoya Biosciences Phenocycler Fusion platform.

**Results:**

Our proteomic analysis revealed the presence of T lymphocyte markers, including CD3, CD45, and CD8, expressing cells and upregulation of immune checkpoint marker PD-L1 within tumor compartments of patients responsive to ICIs, indicative of ‘hot tumor’ phenotypes. We also observed the presence of antigen-presenting cells marked by expression of CD40, CD68, CD11c, and CD163 with upregulation of antigen-presentation marker HLA-DR, in patients responding to ICIs. Transcriptome analysis of TLS and GCs uncovered a marked elevation in the expression of genes related to immune modulation, diverse immune cell recruitment, and a potent interferon response within the TLS structure. Notably, the distribution of TLS-tumor distance was found to be significantly different across response groups (H = 9.28, *p* = 0.026). The proximity of TLSs to tumor cells was found to be a critical indicator of ICI response, implying that patients with TLSs located further from tumor cells have worse outcomes.

**Conclusion:**

The study underscores the multifaceted role of TLSs in modulating the immunogenic landscape of the TME in R/M HNSCC, likely influencing the efficacy of ICIs. Spatially resolved multi-omics approaches offer valuable insights into potential biomarkers for ICI response and highlight the importance of profiling the TME complexity when developing therapeutic strategies and patient stratification.

**Supplementary Information:**

The online version contains supplementary material available at 10.1186/s12967-024-05409-y.

## Introduction

Head and neck squamous cell carcinoma (HNSCC) originates from the nasal/oral cavity, paranasal sinuses, nasopharynx, larynx, and oropharynx, and patients with recurrent and/or metastatic (R/M) disease show poor prognosis [1–3]. Though tobacco use and alcohol are significant risk factors, oropharyngeal HNSCC tumors can be divided into two primary categories based on their human papillomavirus (HPV) infection status [4]. HPV-positive tumors generally have a better prognosis compared to HPV-negative tumors, which are associated with poorer outcomes [4]. Treatment may include surgical, radiotherapy and/or chemotherapy. Upon recurrence or de-novo metastatic disease, the prognosis is poor [5].

Immunotherapy, particularly immune checkpoint inhibitors (ICIs), such as the anti-PD-1 antibodies pembrolizumab and nivolumab, has shown promise in improving outcomes for patients with unresectable R/M HNSCC, however, only a fraction of patients benefit [6–9]. The Keynote-048 study reported that 17% (51 out of 301) of R/M HNSCC patients treated with pembrolizumab monotherapy had an objective response [9]. The immunohistochemistry (IHC)-based PD-L1 expression is used as a predictive biomarker of response to ICIs in HNSCC patients, and its positive score is generally associated with a better response to immunotherapy [10]. Studies have reported that the predictive accuracy of PD-L1 expression improves when assessing the combined positive score (CPS), which evaluates PD-L1 on both tumor cells and infiltrating immune cells, rather than solely the tumor proportion score (TPS), which measures PD-L1 on tumor cells alone [11, 12]. However, evidence indicates that some PD-L1-negative patients still benefit from ICIs, highlighting the limitations of PD-L1 as a solitary predictor for immunotherapy response [10–13]. The tumor microenvironment (TME), with its diverse cellular composition and interactions, which contribute to immunosuppression, tumor growth and therapy resistance, plays a key role in response to ICIs [14–16]. Studies have highlighted the critical role of TLSs within the TME across various tumor types, implying their significance as primary sites for tumor antigen presentation [17, 18]. The presence of TLSs has been linked to enhanced ICI response and improved patient survival [19–21]. TLSs reinforce antitumor responses by supporting dendritic cell antigen presentation, B-cell-mediated immunity, and T-cell activation, maintenance and survival from persistent antigen stimulation [21]. By increasing immune surveillance and promoting the recruitment of distinct immune cell types, the TLSs potentially increase the efficacy of immunotherapeutic agents [5, 22, 23], positioning them as promising biomarkers for predicting treatment response in R/M HNSCC patients. However, research has shown that the presence of TLSs is not the only factor influencing patient responses. The cellular interactions and organizational patterns within TLSs, as well as their proximity to tumor cells, which improves the presentation of tumor-antigen to T cells, all have a significant impact on the efficacy of ICIs [24–26].

**Fig. 1:**
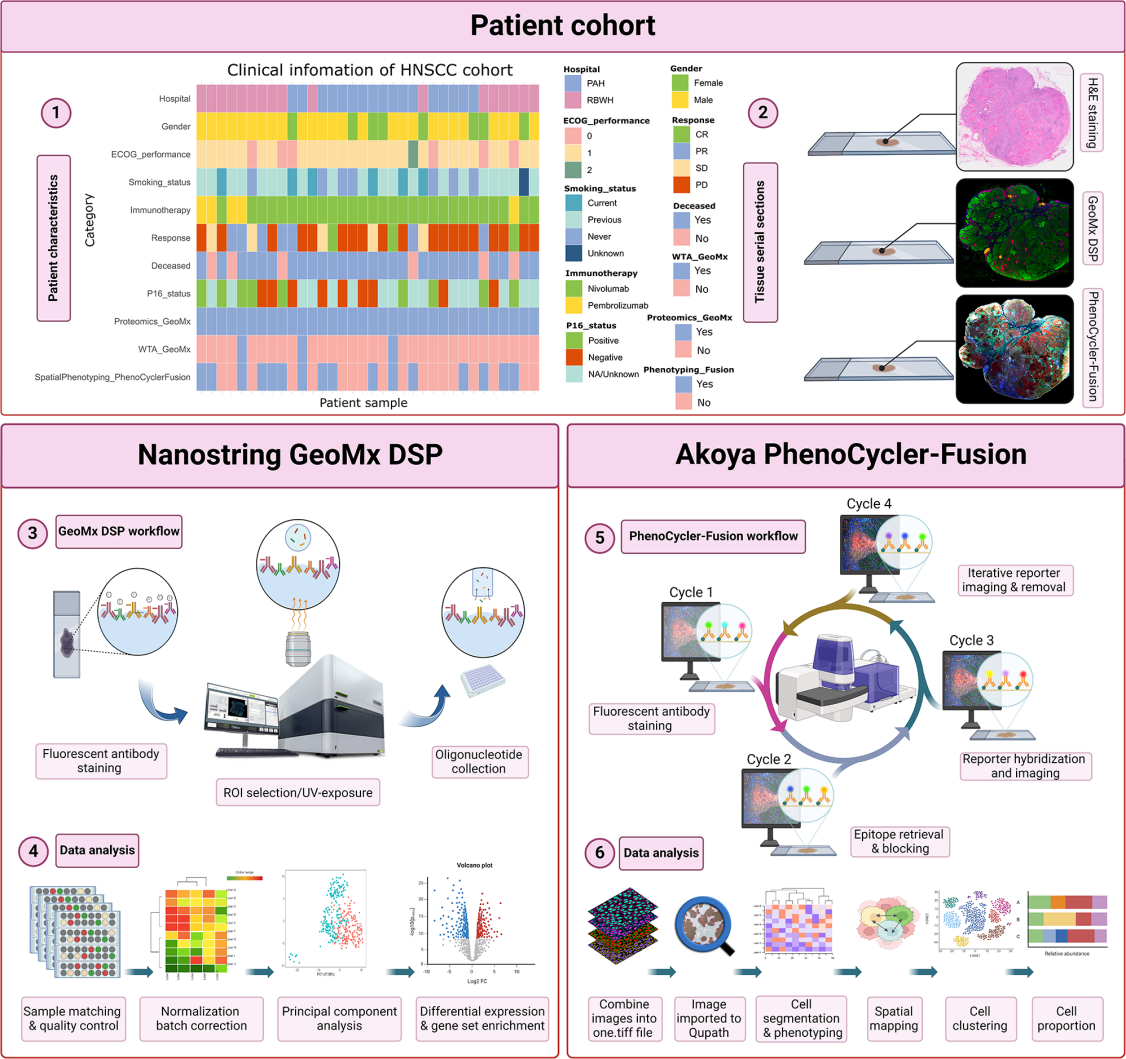
Study scheme. (**1**) Formalin-fixed paraffin-embedded (FFPE) tissue samples were collected prior to therapy from two independent R/M patient cohorts at the Princess Alexandra Hospital (PAH) and Royal Brisbane & Women’s Hospital (RBWH). In each clinical site, 17 samples were determined to be suitable for subsequent spatial analysis. (**2**) Tumor tissue serial sections and hematoxylin & eosin (H&E) staining were provided by the Pathology Queensland. (**3**) Using the Nanostring GeoMx DSP targeted spatial proteomics and transcriptomics were performed across the cohorts. (**5**) Spatial phenotyping of a sub-cohort was performed using the Akoya Biosciences PhenoCycler-Fusion. (**4**, **6**) Data analysis consisted of probe quality control (QC), principal component analysis (PCA), differential expression (DE) and gene set enrichment analysis (GSEA) were conducted and followed by spatial analyses, including cell phenotyping and mapping

The introduction of high-plex spatial profiling and phenotyping technologies has revolutionized our understanding of the TME’s cellular and molecular dynamics [27]. These methodologies enable the detailed examination of immune cell distribution and the functional status of TLSs with unprecedented resolution [28]. In this study, we utilized two advanced spatial profiling technologies, Nanostring Digital Spatial Profiler (DSP) and the Akoya Biosciences PhenoCycler-Fusion, to thoroughly investigate the cellular and molecular characteristic of the R/M HNSCC microenvironment with single-cell and spatial resolution (Fig. 1). Our study found that patients who went on to respond to ICIs had higher infiltrations of immune cell types, primarily those involved in antigen-presentation, within the tumor compartments relative to non-responding patients. We found transcriptome signatures involved in immune modulation and a potent interferon response to be highly expressed in TLSs when compared to normal GCs.

## Materials and methods

### Patient cohort

In this study, we identified a cohort of 41 patients with R/M HNSCC from the Royal Brisbane & Women’s Hospital (RBWH) (Ethics Approval LNR/2020/QRBW/66,744), and an additional 28 patients from the Princess Alexandra Hospital (PAH) (HREC/2022/QMS/89,452), all of whom were determined eligible for inclusion in our study. Among the patients from RBWH, tissue samples were unavailable for 20 individuals, leaving 21 tissue blocks accessible for analysis. We collected formalin-fixed paraffin-embedded (FFPE) tissue specimens from these 21 HNSCC patients prior to immunotherapy. Only 17 of these samples met the criteria for sufficient quality and tissue integrity to proceed with spatial analysis. Similarly, from the PAH cohort, 11 patients had insufficient tissue available, resulting in 17 tissue blocks that were viable for analysis. From these, 17 FFPE tissue samples were collected and found to be suitable for our subsequent spatial analysis. Pathology Queensland undertook the preparation of serial sections and performed hematoxylin and eosin (H&E) staining. To ensure the exclusion of non-neoplastic epithelial cells, expert pathologists demarcated tumor and stromal regions. ICI administered were pembrolizumab and nivolumab for the RBWH cohort and nivolumab for the PAH cohort. All patients were categorized based upon response to therapy according to Response Evaluation Criteria in Solid Tumors, version 1.1, (RECIST 1.1), which includes complete response (CR), partial response (PR), stable disease (SD), and progressive disease (PD). Tumour PD-L1 scoring was not available at the time of this study as combined proportion score (CPS) was not routinely tested at the time of patient treatment.

### Nanostring GeoMx digital spatial profiler

The FFPE tissue sections from 34 patients were stained and processed for proteomics analysis using the Nanostring Digital Spatial Profiler (DSP) according to the manufacturer’s instructions at the Translational Research Institute (TRI) and Queensland University of Technology (QUT), Brisbane, Australia. Using the Nanostring immuno-oncology (IO) panel, which comrises 71 oligonucleotide-conjugated antibodies for immune cell profiling core, immune cell typing and activation modules, cell death, IO drug target, PI3k/AKT signaling, and pan-tumor pathways, the tissue sections were analyzed to investigate the protein expression of immune and tumor biomarkers (Supplementary Table 1). Additionally, to perform the whole transcriptome analysis, the FFPE tissue section was collected from one R/M HNSCC patient. The Nanostring Whole Transcriptome Atlas (WTA) panel, comprising over 18,000 genes, was utilized and the tissue section was processed and analyzed based on the manufacturer’s instructions. Morphology markers for both proteomics and transcriptomics assays included pan-cytokeratin (PanCk), CD45, and SYTO-13 to distinctly identify tumor cells, immune cells, and nuclei, respectively. Subsequently, the demarcation of tumor and stromal compartments was accomplished by gating PanCk positive and negative regions. Quantification of antibody barcodes and the sequencing of target oligonucleotides were performed using the Nanostring nCounter technology and the Illumina’s NovaSeq 6000, respectively, following the manufacturers’ guidelines. The final stage of the process involved the utilization of the DSP analysis suite, where External RNA Controls Consortium (ERCC) normalization and quality control (QC) procedures were executed. These steps were critical in refining the data output, thereby rendering it suitable for subsequent bioinformatic analyses.

### Nanostring GeoMx DSP data analysis

The GeoMx DSP proteomics data consists of 468 ROI samples from 34 patients across 2 cohorts. Each sample ROI was quantified for 71 protein markers, 3 housekeeper proteins (Histone H3, GAPDH, S6) and 3 background IgG controls (Rb IgG, Ms IgG2a, Ms IgG1). The data analysis was performed using probeQC counts from Nanostring DSP analysis suite. Quality control and data processing were conducted based on the Bioconductor R package *standR (v1.6)* [29] workflow to perform quality control, data normalization and batch correction. Initially, filtering was conducted to exclude regions of interest (ROIs) with low surface area and low nuclei count, and to exclude genes that were not expressed or had low expression in more than 90% of ROIs.

For data analysis, the log2-transformed count per million (logCPM) value was used to account for variation from library size. The scaled count data was subjected to the relative log expression (RLE) and principal component analysis (PCA) to investigate overall distribution, underlying factors from the experimental design and the unwanted batch effects. Subsequently, to remove batch effect between the experiments of two cohorts in the protein data, 30 negative control markers between batches were first selected using the *findNCGs* function, followed by using the RUV4 batch correction method [30] within the *geomxBatchCorrection* function (Supplementary Fig. 1). Subsequently, the efficacy of the correction was assessed through a reapplication of RLE and PCA analyses (Supplementary Fig. 2).

To perform differential expression (DE) analysis, R packages *edgeR (v3.34.0)* and *limma (3.48.0)* were applied to the count data following the *standR* workflow [29]. *duplicateCorrelation* from the *limma* package was first used to calculate the consensus correlation across patients to account for patient variation as a random effect. DE was then performed using linear models, which incorporated experimental factors as predictors. The combination of universal dispersion, affecting all genes, and marker-specific dispersion was used for the variations in marker expressions. Using an empirical Bayes approach, the variation for each marker was estimated by combining data from all other markers to calculate both universal and marker-specific variations. The factors of interest in this study includes responses to treatment (i.e., *CR, PR, SD, PD*) with the following comparisons made: (A) *CR vs. PD* and (B) Disease control rate i.e., *CR + PR + SD vs. PD*.

The GeoMx DSP transcriptomics data was generated using the Nanostring GeoMx WTA panel on the GeoMx DSP. The data consisted of RNA abundance measurements of over 18,000 genes with measurements made on regions of interest across 3 patients’ tissue sections, however, one sample was eligible for our TLS analysis. In total, 9 ROIs were analyzed from four GCs and one TLS. The RNA data was processed using the *standR* workflow: Sample filtering to exclude ROIs with low detection count < 350,000, low nuclei count < 250 and those with high percentage of low expressing genes (> 3%) did not remove any ROI. No genes were removed from gene filtering to exclude low expression genes. logCPM counts was assessed using RLE plots and PCA to investigate factors contributing to the variation in the data and to identify batch effects, resulting in the removal of 2 ROIs from a GC as outliers. The filtered data were normalized using the trimmed mean of M-values (TMM) method using all genes in the panel. DE analysis was conducted using the R package *edgeR* based on the *voom-limma pipeline with sample weights.* The factor of interest in this analysis is the category (TLSs or GC). The *edgeR::voomLmFit* function was used to fit a linear model with “category” as a covariate and with the comparison of interest investigated as *TLS vs. GC.*

For both the RNA and protein analyses conducted in this study, the linear modelling uses a robust empirical Bayes moderated t-statistic and the Benjamini–Hochberg procedure was applied for multiple testing adjustments (adjusted p-value, FDR of < 0.05).

### Akoya Biosciences PhenoCycler–Fusion staining and cell segmentation

PhenoCycler-Fusion (Akoya Biosciences, USA) staining and whole-slide imaging of 16 FFPE samples was performed according to the manufacturer’s instructions as described previously [31]. Samples were profiled in collaboration with Akoya Biosciences’ Technology Access Program and the Queensland Spatial Biology Centre (QSBC, Wesley Research Institute). Antibody targets, conjugated barcodes, and corresponding fluorophores are listed in Supplementary Table 2. Following image acquisition, the resulting qptiff file was opened in QuPath (v0.5.0) [32] where each marker was qualitatively assessed for reliable and robust staining. Cell segmentation was then performed by applying the StarDist (v0.8.5) [33, 34] model *dsb2018_heavy_augment* to the DAPI image. Each cell’s spatial coordinates and median intensity of each marker were then exported from QuPath.

### PhenoCycler-Fusion data analysis

#### Cell typing

The Qupath output is converted into the Anndata [35] format to conduct additional spatial analysis. The low median DAPI signal cut-off is used to eliminate artificial nuclei; tiny and large nuclei are eliminated by taking into account a tolerance of 1% on either side of the size distribution. Then, the expression matrices are transformed with arcsinh (cofactor 150), scaling within markers, and scaling across cells. After performing PCA and batch correction, phenograph clustering was conducted using sixteen markers—*CD11c, CD20, CD21, CD31, CD34, CD3e, CD4, CD45, CD45RO, CD68, CD8, HLA-A, HLA-DR, Ki67, Pan-Cytokeratin*, and *Podoplanin*.

### Tertiary lymphoid structure (TLS) quantification

To assess and quantify TLSs, our methodology began with the precise demarcation of peritumoral areas, defined as regions extending up to 1000 μm from the tumor nest boundary [20, 22]. This specific boundary selection is based on existing literature that emphasizes the exclusive formation of TLSs within these defined perimeters and distance from the tumour [18, 20]. We identified TLSs either within the tumoral or peritumoral areas [18]. Employing PhenoCycler-Fusion technology, we then identified and characterized dense lymphoid aggregates of CD20^+^ B cells within the intratumor or peritumoral zones as TLSs. This method enabled the detection of TLSs in 10 out of the 16 analyzed whole tissue samples [5].

### TLS distance analysis

The distance between TLSs and the closest tumor cells was measured using the distance measurement between the TLS and the Tumor annotations in QuPath (v0.5.0) [32], and then the mean value of distance in each response group was taken into account. The Kruskal-Wallis test was used to compare the distributions of TLS to tumor distances in different response groups. A p-value of 0.05 was used to determine statistical significance between the distance distributions of the groups. Next, Dunn’s test was employed to conduct pairwise comparisons while considering a Bonferroni correction for multiple comparisons [24, 35].

## Results

### R/M HNSCC patient characteristics

This study examined two independently collected cohorts of 69 patients with R/M HNSCC from July 2015 to December 2021, contributing 34 whole-slide specimens suitable for spatial analysis. Among those patients, 26 (76%) were male and 8 (24%) were female. The patients were categorized into four groups based on their therapeutic responses, as defined by the RECIST 1.1 criteria, which included *n* = 3 (9%) complete response (CR), *n* = 7 (21%) partial response (PR), *n* = 5 (15%) stable disease, and *n* = 19 (55%) progressive disease. In terms of HPV status, the distribution was as follows: *n* = 9 (26%) patients were HPV-positive, *n* = 9 (26%) patients were HPV-negative, and the remaining *n* = 16 (48%) patients were classified as either not applicable or unknown. The association of cohort characteristics with best response was shown in Supplementary Fig. 3. The clinicopathological characteristics of the patient cohorts are shown in Table 1.

**Table 1:** R/M HNSCC cohort characteristics

Patients’ characteristics	
**Characteristics**	**All patients** ***(N = 34)***
**Age, median (range)**	66 (29–83)
**Status**	
Alive	6 (18%)
Deceased	28 (82%)
**Gender**	
Male	26 (76%)
Female	8 (24%)
**Smoking status**	
Current/former smokers	26 (76%)
Non-smokers	7 (21%)
Unknown	1 (3%)
**ECOG performance status**	
0	6 (18%)
1	27 (79%)
2	1 (3%)
**Tumor location**	
Oropharynx	19 (55%)
Oral cavity	11 (33%)
Larynx	3 (9%)
Hypopharynx	1 (3%)
**Immunotherapy**	
Pembrolizumab monotherapy	3 (9%)
Pembrolizumab + chemotherapy	1 (3%)
Pembrolizumab + IDO1 inhibitor	1 (3%)
Nivolumab monotherapy	29 (85%)
**Best response**	
Complete response (CR)	3 (9%)
Partial response (PR)	7 (21%)
Stable disease (SD)	5 (15%)
Progressive disease (PD)	19 (55%)
**Oropharynx p16 status**	
Positive	9 (26%)
Negative	9 (26%)
Unknown (not tested)	16 (48%)

### Differentially expressed protein biomarkers identified using Nanostring GeoMx DSP

**Fig. 2:**
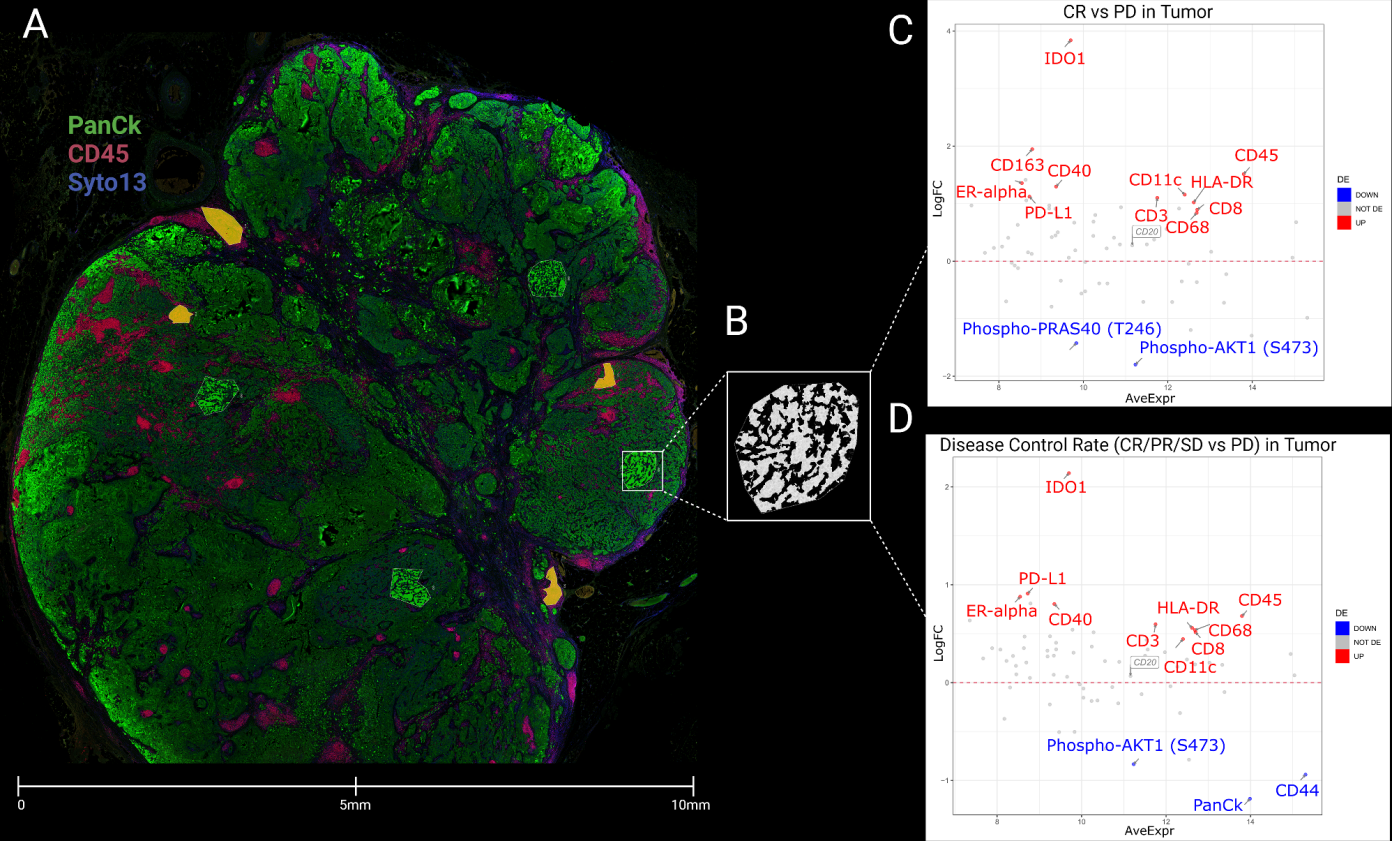
Differential protein expression in patients with different response groups. (**A**) Spatial proteomics profiling was conducted on tissue samples from R/M HNSCC tumor tissues. Demarcation of tissues was achieved through masking PanCk + and PanCk- regions to delineate tumor and stromal compartments, respectively. The morphology markers included PanCk (green), CD45 (red), and SYTO 13 (blue) for the tumor cells, immune cells, and nucleus, respectively. Segmentation of tumors focused on regions of interest (ROIs) to distinctly identify the Tumor mask in green and the Stromal mask in yellow. (**B**) Utilizing the PanCk+/- feature, masks were generated to liberate barcodes for digital counting via the Nanostring nCounter platform. (**C**) MA plots of Mean Expression (AveExpr) vs. fold change (logFC) visualize the expression of protein biomarkers within tumor compartments in patients with complete response (CR) versus patients with progressive disease (PD). (**D**) MA plots of Mean Expression (AveExpr) vs. fold change (logFC) visualize the expression of protein biomarkers within tumor compartments in patients with disease control status (CR/PR/SD) versus patients with PD. Color coding represents markers that are not differentially expressed (gray), significantly upregulated (red), and downregulated (blue), based on a false discovery rate (FDR) of < 0.05 following multiple testing adjustments

Using the Nanostring GeoMx DSP, we investigated the protein expression profiles within tissue samples from 34 patients with R/M HNSCC tumors, prior to ICI treatment (Fig. 2A). We segmented the tumor and stromal compartments by applying masks to PanCk-positive and -negative regions, respectively (Fig. 2B). Subsequently, we assessed the differentially expressed (DE) proteins within these compartments among patients with different response groups based on the RECIST 1.1 criteria.

Initially, our focus was on the DE proteins across different response groups, including patients with CR (*n* = 3), PR (*n* = 7), SD (*n* = 5), and PD (*n* = 19). Our findings indicated a significantly (*p* < 0.05) higher expression of protein biomarkers associated with the presence and activity of immune cells, including CD45, CD3, CD68, CD163, CD11c, CD8, HLA-DR, CD40, PD-L1, and IDO1, in the tumor compartments of patients with CR compared to those with PD (Fig. 2C). Moreover, we found a decreased expression of protein biomarkers involved in the survival and proliferation of tumor cells in the tumor compartments of CR patients relative to those with PD (Fig. 2C). We then classified those 34 patients into two groups based on their disease control status. Patients with CR, PR, and SD were grouped as responders (*n* = 15), while those with PD were categorized as non-responders (*n* = 19). In this classification, the DE proteins mirrored our earlier analyses, indicating similar differential expression of the aforementioned protein biomarkers (Fig. 2D). However, it should be noted that data generation was limited because these protein biomarkers were only collected and profiled from user-defined regions of interest (ROIs). This approach contributes to a potential oversight in capturing the full spectrum of tumor and TME biology. Specifically, B-cell characteristics and the presence of TLSs might be missed if these specific areas are not included in the ROIs profiled.

### Whole transcriptome analysis using Nanostring GeoMx DSP

**Fig. 3:**
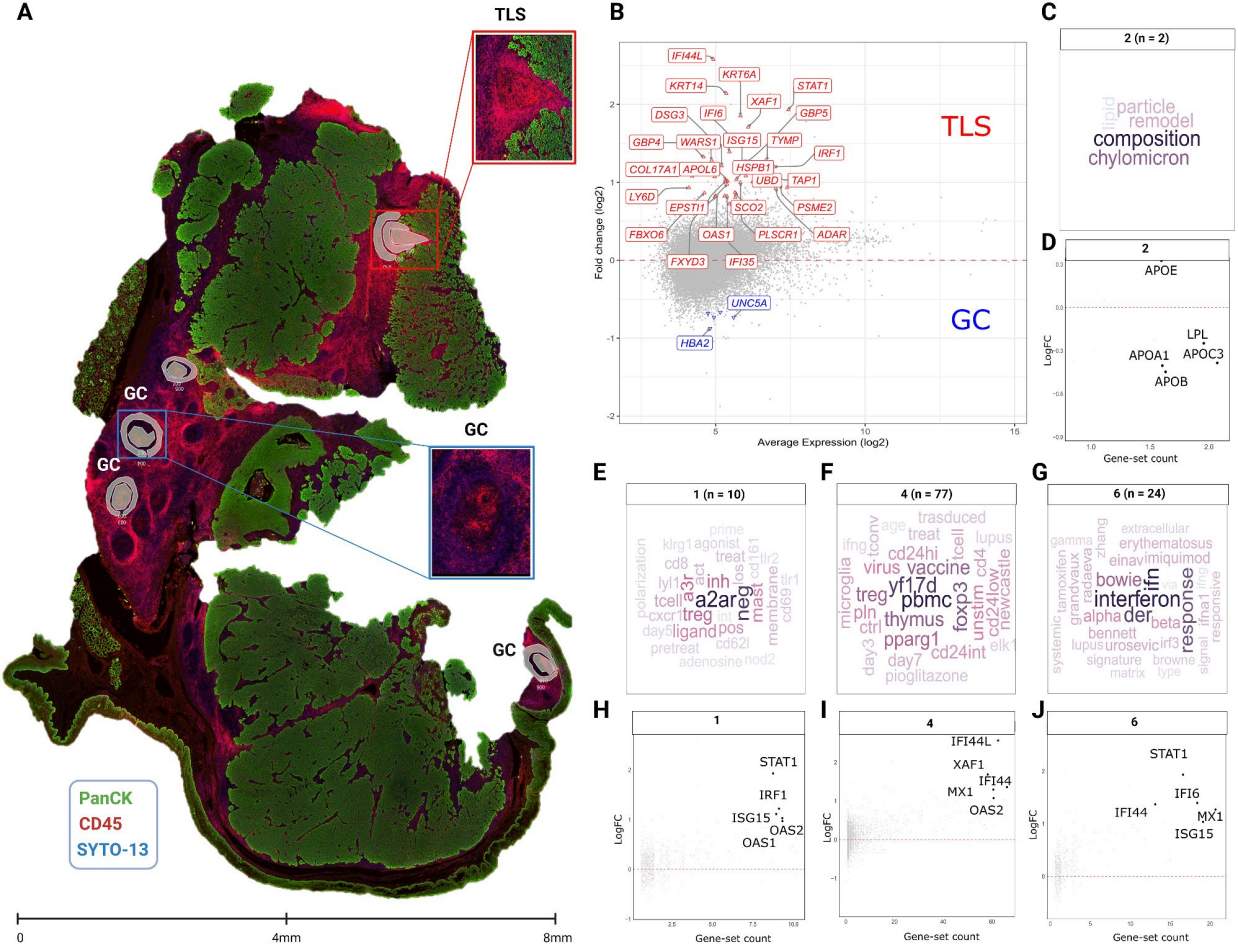
Visualization of significantly enriched gene sets different comparisons. (**A**) Spatial transcriptomics profiling was conducted on an R/M HNSCC tissue sample. Demarcation of the tissue was achieved through masking PanCk + and PanCk- regions to delineate tumor and stromal compartments, respectively. The morphology markers included PanCk (green), CD45 (red), and SYTO 13 (blue) for the tumor cells, immune cells, and nucleus, respectively. Utilizing the CD45+/- feature, masks were generated to liberate barcodes for sequencing via the Illumina’s NovaSeq 6000 platform. (**B**) The differential gene expression in TLS regions against GCs visualised as mean transcript expression (AveExpr, in log2) versus fold change (logFC, in log2). Color represents markers that are not differentially expressed (gray), significantly upregulated (red), or downregulated (blue), based on a false discovery rate (FDR) of < 0.05 following multiple testing adjustments. (**C**, **E**, **F**, and **G)** Gene-set clusters identifying dominant biological themes (**E-G**) upregulated or (**C**) downregulated in the comparison TLS vs. GCs with gene-set names depicted as representative wordclouds. Clusters 1,2,4, and 6 representing distinct biological themes were identified in each comparative analysis. (**D**, **H**, **I**, and **J**) The corresponding gene statistics (i.e. fold change in log2) within the gene sets clusters are plotted against the number of gene sets in the cluster to which the gene is differentially expressed

Following the identification of upregulated protein biomarkers, which were largely associated with immune activity and antigen presentation, we conducted a whole transcriptome analysis on TLS and GC structures to identify any significant differential pathways between these entities. First, we measured gene expression from 9 selected ROIs from one TLS and three GCs (Fig. 3A), then performed a DE analysis (Fig. 3B). This analysis revealed 30 genes differentially expressed between the TLS and GCs, with 28 upregulated and 2 downregulated (Fig. 3B). Subsequently, we applied Gene Set Enrichment Analysis (GSEA) to the DE genes (Fig. 3C-J). When comparing TLS and GCs, the gene set similarity network was represented as a network (Supplementary Fig. 4A, B). The visualization of upregulated gene sets in the comparison between TLS and GCs uncovered three significant clusters, representing immune-modulating activities, the presence of a variety of immune cell types, and a potent interferon response indicative of an antiviral-like immune reaction (Fig. 3E-G). Cluster 1 highlighted an elevated activity of the adenosine A2A receptor (A2AR), indicating an immunomodulatory function within the TLS (Fig. 3E, H). Cluster 4 revealed the presence of diverse immune cell types in the TLS as compared to GCs (Fig. 3F, I), and Cluster 6 underscored an increased level of interferon signaling-related genes, fostering a robust antiviral-like immune response within the TLS (Fig. 3G, J). In contrast, the visualization of downregulated gene sets in the TLS versus GCs identified a diminished lipid metabolism pathway in Cluster 2 (Fig. 3C, D), as evidenced by genes such as APOE, LPL, APOC3, APOA1, and APOB (Fig. 3D).

### Spatial analysis of TLSs

**Fig. 4:**
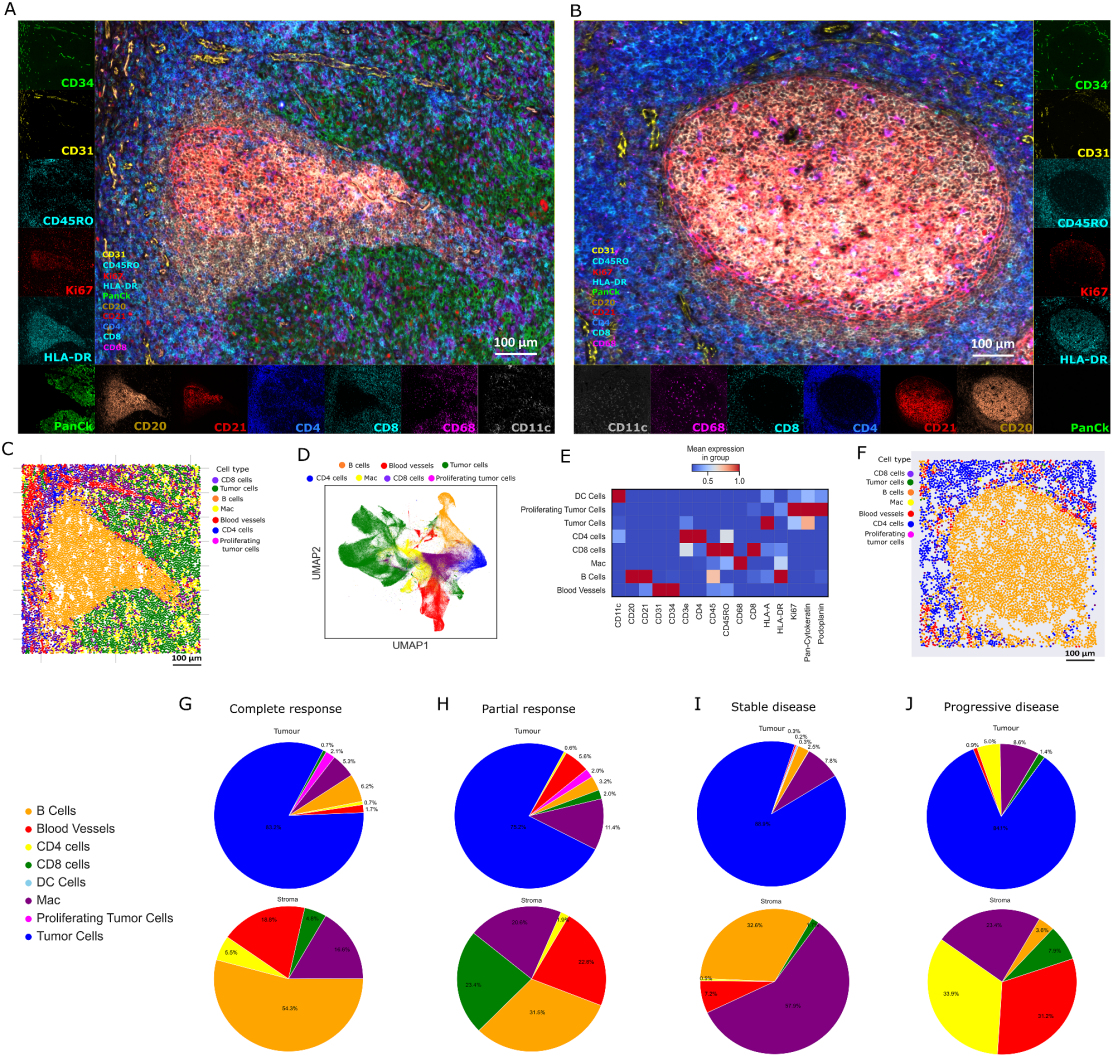
Cell types. (**A**, **B**) Multiplex immunofluorescence imaging of key immune cell markers within TLS at single-cell resolution. The morphology markers included CD31 (yellow) and CD34 (green) for blood vessels, CD45RO (cyan) for memory T cells, Ki67 (red) for cell proliferation, HLA-DR (cyan) for antigen presentation, PanCk (green) for tumor cells, CD20 (brown) and CD21 (red) for B cells, CD4 (blue) for CD4^+^ T cells, CD8 (cyan) for CD8^+^ T cells, CD68 (purple) for macrophages, and CD11c (grey) for dendritic cells. (**C**) Representative image of cell types in the TLS structure. (**D**) Annotated clusters were merged into 7 cell types. (**E**) Heatmap indicating clustering of cell types based on markers. (**F**) Representative image of cell types in the GC structure. (**G**, **H**, **I**, and **J**) Representative images of cell proportions within the tumor and the stromal compartments of patients with different response group, including, CR, PR, SD, and PD, respectively

Based upon our proteomics and transcriptomics findings which revealed increased immune infiltration and activity in TLS structures, we next aimed to characterize the TLS and GC architecture. The above-mentioned transcriptomics and proteomics findings were based on regions of interest within the tissue, thus, to have a more comprehensive understanding of the cellular organization and interactions of the TLS and GC cell populations, the whole-slide phenotyping of the tissue samples was performed using the PhenoCycler-Fusion platform at single-cell resolution (Fig. 4A, B). This included the identification of cell types and their interactions. By using a 16-plex antibody panel and applying unsupervised spatial clustering of immune cell markers (Fig. 4E), we phenotyped cell types within the TLS (Fig. 4C) and GC (Fig. 4F) in 10 out of 16 analyzed whole tissue samples. Clusters were annotated and merged into 7 cell types (Fig. 4D).

**Fig. 5:**
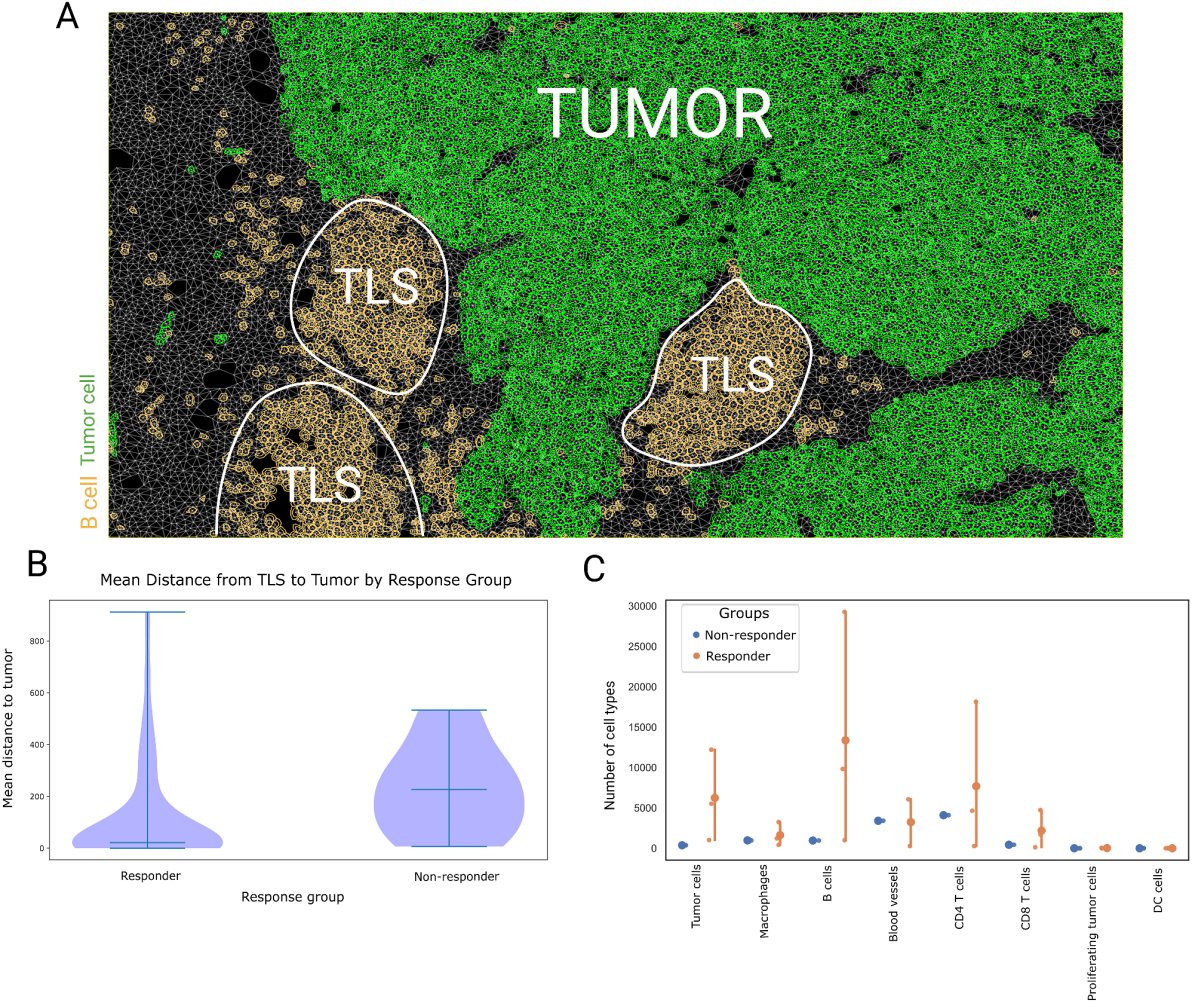
Cellular interaction and distance analysis. (**A**) Representative field of view of enriched TLS: tumor interactions. Morphology markers included CD20 (orange) for B cells and PanCk (green) for tumor cells. (**B**) Violin plot representing the mean distance of TLS to the closest tumor cell in each response group. (**C**) Combined strip and point plots visualizing the number of cell types per response group. Responder group representing patients with CR/PR/SD, and Non-responder representing a patient with PD

We further investigated functional cell-cell relationships to assess the distance between each TLS and the closest tumor cell among different response groups. This aimed to uncover any relationship between the spatial proximity of TLSs to tumor cells and response to ICIs (Fig. 5A). The test found a significant difference in the distributions of TLS-tumor distance across the groups (H = 9.28, *p* = 0.026). Subsequently, pairwise comparisons using Dunn’s test showed significant difference between responders versus non-responders (*p* < 0.05) (Fig. 5B). Our findings revealed that a greater distance between TLSs and tumor cells correlated with a lower response to ICI (Fig. 5B). These data suggest that the spatial positioning of a TLS structure relative to tumor cells may play a crucial role in determining the outcome of ICIs, emphasizing the importance of considering tumor microenvironment architecture when developing treatment strategies. Additionally, we showed that the number of B cells within 100 μm outside of the TLS structures are higher in patient responders than non-responders (Fig. 5C).

## Discussion

The prognosis for patients with head and neck squamous cell carcinoma (HNSCC) in the recurrent and/or metastatic (R/M) setting is generally poor [5–8]. ICIs have emerged as promising interventions for R/M HNSCC patients, demonstrating efficacy as both monotherapy and in combination with chemotherapy [8, 9], however, only a subset of patients benefit from this therapy [5, 9]. The complex interactions of tumor cells within the tumor microenvironment (TME), alongside immune cells, have been identified as critical determinants of response to ICIs [14, 15, 36]. A deeper understanding, therefore, of the cellular interactions within the TME, influence these interactions, may improve patient stratification, potentially identifying individuals more likely to respond to ICIs [2, 14]. One such key interaction within the TME that determines response to therapy is the presence of mature tertiary lymphoid structures (TLSs), defined by the presence of B cell follicles, follicular dendritic cells and a mixture of CD4^+^ and CD8^+^ T cells within the intra- or peritumoral regions [37]. TLSs function as a major source of antigen presentation, contributing to the maintenance and survival of a self-renewing stem-like memory CD8^+^ T cells that undergo proliferative burst upon immune checkpoint inhibitors, and show more robust cytotoxicity antitumor function [19–21, 38]. This study delves into the intricate dynamics of TLSs within the TME of R/M HNSCC patients in order to unravel their impacts on immunotherapeutic outcomes.

In the present study, we found an increased infiltration of immune cell types, primarily antigen-presenting cells, within the tumor compartments of patients who went on to respond to ICI compared to non-responders. This underscores the importance of a distinct immune presence in determining the efficacy of treatment interventions. The significant upregulation of immune cell markers CD3, CD45, CD8, and PD-L1 in the tumor compartments of patients responsive to ICIs highlights the vital role of T cell-mediated immunity, suggesting the presence of a ‘hot tumor’ phenotype. These findings are consistent with the literature, which suggests that elevated T-lymphocyte infiltration could be a biomarker for ICI response in R/M HNSCC patients [39, 40].

Furthermore, the spatial proteomics analysis of tumor compartments between patients with disease control status (CR/PR/SD) and patients with progression (PD) revealed that the former group had higher expressions of immune markers such as CD20 (though not statistically significant), CD40, CD68, HLA-DR, CD11c, and CD163. These markers, characteristic of antigen-presenting cells, imply a potent mechanism of antigen presentation within the TME of patient responders. These findings are supported by existing studies that highlight the significant role of TLS structures in orchestrating immune responses, especially by presenting tumor-specific antigens to T cells [21, 41, 42]. By providing antigenic stimulation, TLSs maintain the survival of stem-like memory CD8^+^ T cells, contributing to a more robust antitumor response [38, 43].

To better understand this, we performed a comprehensive whole transcriptome analysis with a focus on TLS and germinal centre (GC) structures. We identified a significantly elevated expression of specific immune signatures in the TLS, indicating immune modulation, the involvement of various immune cell types, and an interferon-stimulated antiviral-like immunity, in contrast to GC. These findings are critical as they validate that TLSs actively participate in increasing the immunogenicity of the TME [21, 44]. A study by Di Caro et al., showed that the presence of TLS in the colorectal cancer (CRC) was associated with T lymphocyte infiltration and recruitment [44]. The upregulation of these pathways suggests that TLSs function as a hub for immune cell recruitment and activation, allowing for a broad and potent antitumor immune response [17]. The presence of TLSs in tumor tissues of cancer patients is associated with ICI response, and their presence prior to treatment could serve as a predictor biomarker of response [17, 45–47]. Additionally, the active interferon response within the TLS structures highlights the ability of TLSs to engage in antiviral-like immune reactions [48]. These characteristics are crucial in the context of viral-associated HNSCC, especially those driven by HPV [48]. Our findings also demonstrated a downregulation of gene signatures related to lipid metabolism in the TLS compared to GCs, suggesting metabolic reprogramming within the TME of HNSCC tumors [49–51].

Our proteomics and transcriptomics findings underscore the pivotal function of TLSs in supporting an environment that promotes antitumor immunity. Following these results, our spatial analysis provides an important perspective on the functional dynamics of TLSs within the TME, particularly in terms of their spatial arrangement and interaction with tumor cells.

A key finding from our study was that, measuring the distance between TLSs and tumor cells provided critical insights into how the spatial organization of the TME influences ICI outcomes. In this study, we found that patients with a less favorable response to ICIs had TLSs located further away from the tumor cells. These findings indicate that TLSs are not randomly distributed but are purposely positioned in proximity to tumor cells. Similarly, Pfannstiel et al. found that a close distance between TLSs and tumor invasive front was significantly associated with an inflamed immune phenotype and improved patient survival in muscle-invasive bladder cancer [26]. This spatial correlation demonstrates a synergistic relationship between immune activation molecular signatures and their spatial presentation within the TME, implying that cancer treatment success is dependent on both the presence of TLSs and their spatial localization.

### Study limitations

Our study has a number of limitations, which include the use of a region of interest (ROI)-based assay (GeoMx DSP) and limited size of the patient cohort. Due to the paucity of TLS structures in the tissues able to be collected, our transcriptomic analysis was limited to a single sample where we compared defined germinal centre biology to that of TLS. Additional samples would have allowed these measurements to be made across a greater diversity of TLS maturity for association with therapy outcome. The absence of a standardized approach for TLS quantification presents a methodological challenge. TLS identification and quantification methods differ across studies [25], making comparisons difficult and potentially preventing the development of a comprehensive understanding of TLS function in cancer immunotherapy. Additionally, the markers used in multiplex imaging were limited to basic cell linages and did not provide insights into how their functional characteristics may associate with TLS biology. Addressing these limitations in future research will be critical to improving our understanding of TLS dynamics and their implications for better therapeutic outcomes in patients with R/M HNSCC.

## Conclusion

This study revealed a complex interaction within the TME, where the molecular and cellular compositions of TLSs are fundamentally linked to their spatial arrangement relative to tumor cells. This intricate interplay is critical for understanding the mechanisms behind the variable responses to ICI in R/M HNSCC patients. The application of sophisticated spatial profiling and phenotyping technologies has been instrumental in gaining these insights, allowing us to precisely map the localization of TLSs and delineate their cellular architecture. These methodologies have provided the essential framework for connecting our molecular insights with the physical structure of the TME. This emphasizes the significance of spatial arrangements and detailed cellular composition of TLSs in converting biological cues into potent antitumor immune responses.

## Electronic supplementary material

Below is the link to the electronic supplementary material.

Supplementary Material 1

Supplementary Material 2

Supplementary Material 3

Supplementary Material 4

Supplementary Material 5

## Data Availability

Geo accession numbers: GSE259279 AND GSE259280.
