# Supplementary figures and images for: Spatial dynamics of tertiary lymphoid aggregates in head and neck cancer: insights into immunotherapy response
Source: J Transl Med. 2024 Jul 24;22:677. doi: 10.1186/s12967-024-05409-y (PMC11267849; doi:10.1186/s12967-024-05409-y)

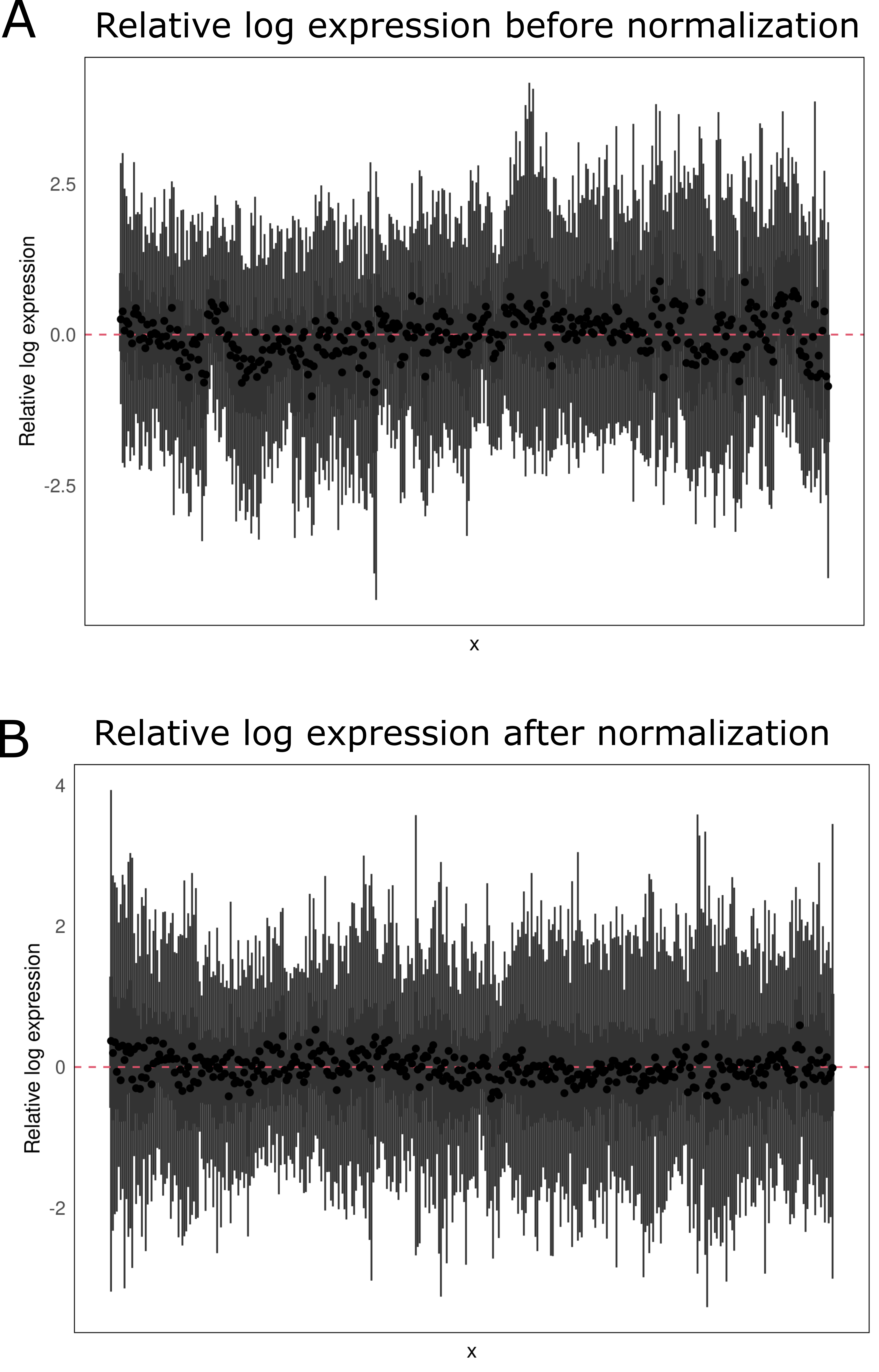

Supplement: Supplementary file 1 — Supplementary Material 1 [file 12967_2024_5409_MOESM1_ESM.png]

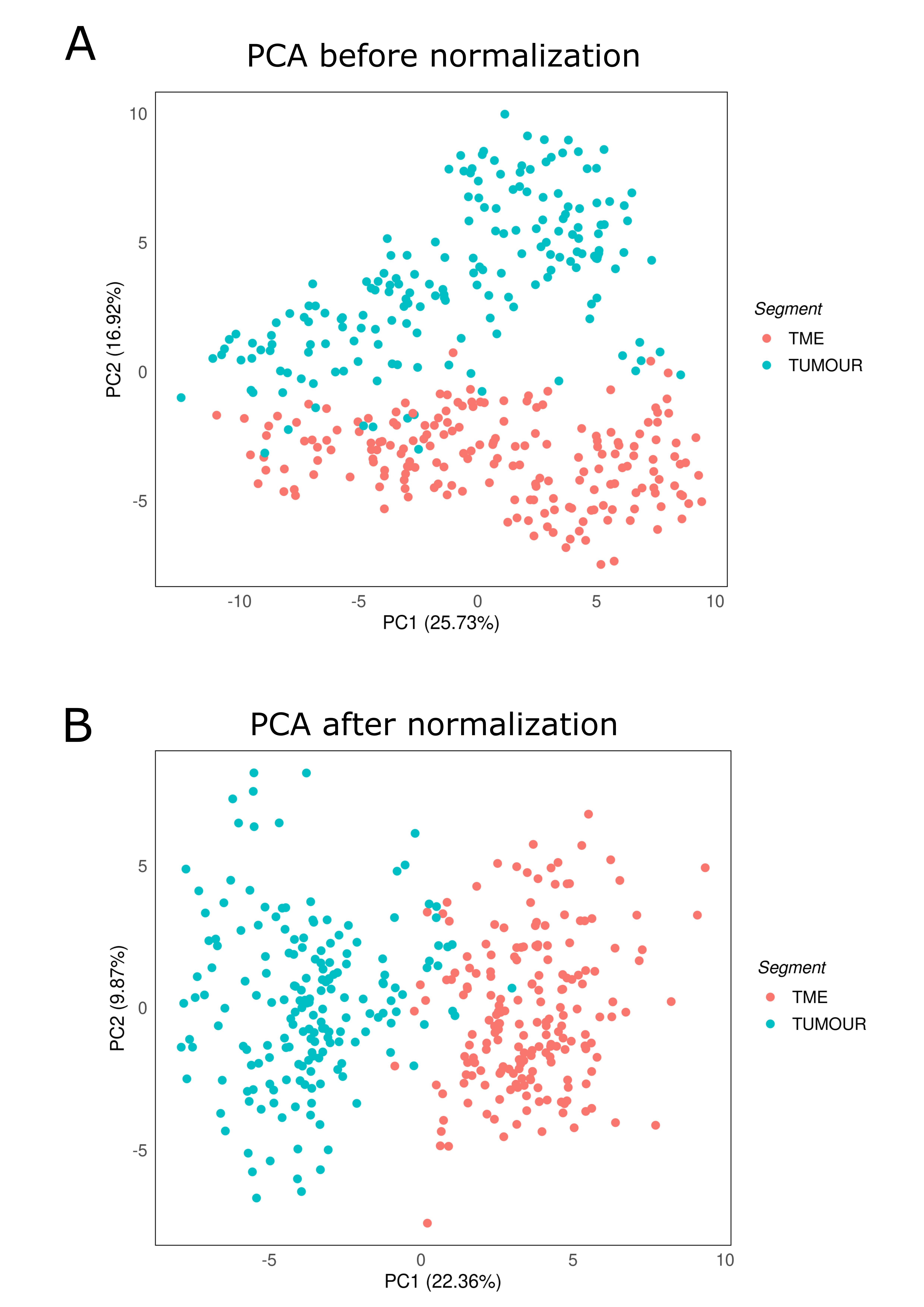

Supplement: Supplementary file 2 — Supplementary Material 2 [file 12967_2024_5409_MOESM2_ESM.png]

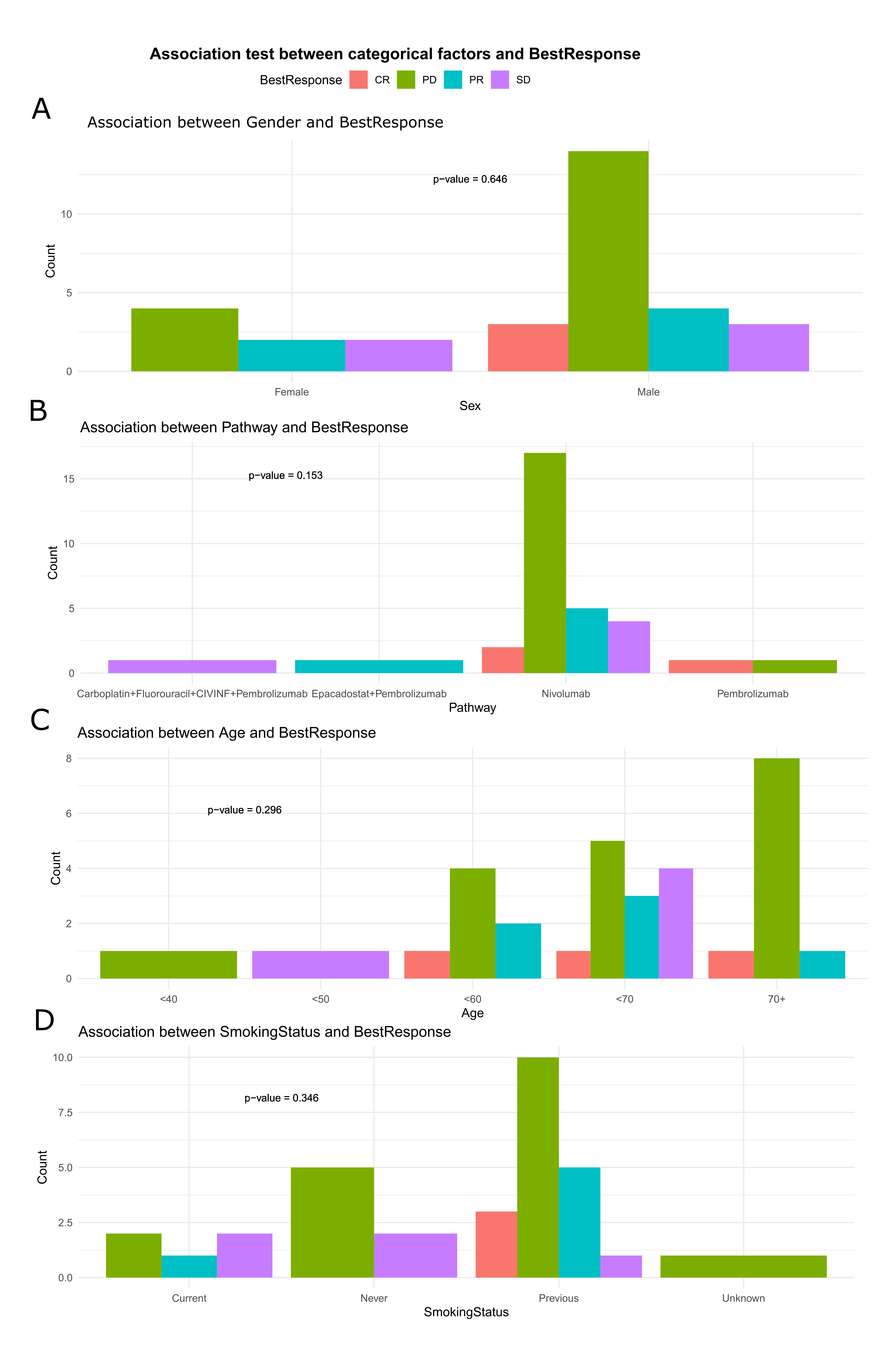

Supplement: Supplementary file 3 — Supplementary Material 3 [file 12967_2024_5409_MOESM3_ESM.png]

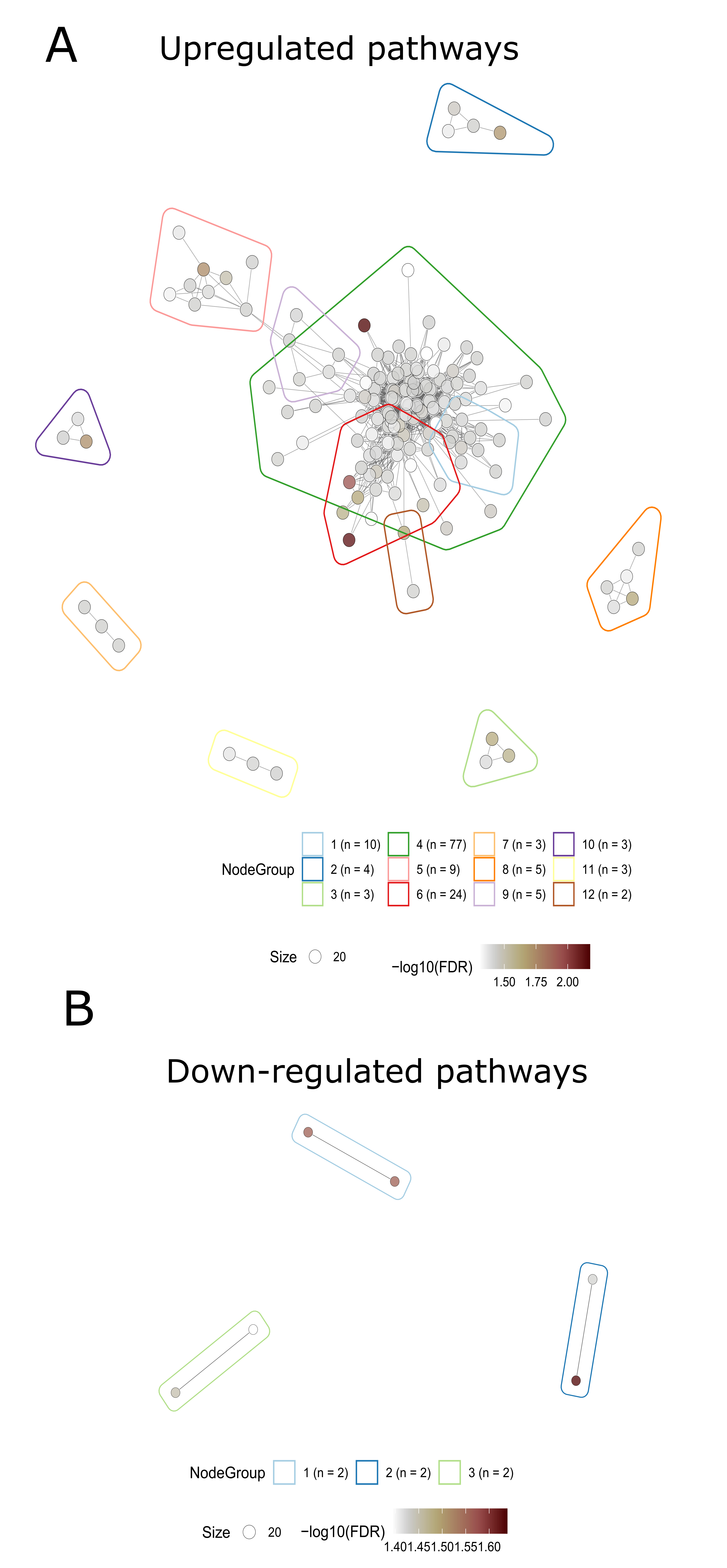

Supplement: Supplementary file 4 — Supplementary Material 4 [file 12967_2024_5409_MOESM4_ESM.png]
