# Supplementary material for: Spatial dynamics of tertiary lymphoid aggregates in head and neck cancer: insights into immunotherapy response
Source: J Transl Med. 2024 Jul 24;22:677. doi: 10.1186/s12967-024-05409-y (PMC11267849; doi:10.1186/s12967-024-05409-y)
Supplement: Supplementary file 5 — Supplementary Material 5 [file 12967_2024_5409_MOESM5_ESM.docx]

*Supplementary Table 1. Nanostring immune-oncology panel*

| **Immune Cell Profiling** | **Immuno-oncology Drug Target** | **Immune Activation Status** | **Cell Death** | **PI3K/AKT signalling** | **Immune Cell Typing** | **Pan-Tumour** |
| --- | --- | --- | --- | --- | --- | --- |
| **Beta-2-microglobulin** | 4-1BB | CD127 | BAD | Pan-AKT | CD14 | BCL-2 |
| **CD11c** | ARG1 | CD25 | BCL6 | MET | CD163 | EpCAM |
| **CD20** | B7-H3 | CD27 | BCLXL | Phospho-AKT1 (S473) | CD34 | ER alpha |
| **CD3/ CD45** | GITR | CD40 | BIM | Phospho-GSK3B (S9) | CD45RO | HER2/ERBB2 |
| **CD4/ CD8** | IDO1 | CD44 | CD95/Fas | Phospho-Tuberin (T1462) | CD66b | MART1 |
| **Histone H3/ S6** | LAG3 | CD80 | GZMA | Phospho-GSK3A (S21)/ Phospho-GSK3B (S9) | FAP-alpha | NY-ESO-1 |
| **Ms IgG2a/ Ms IgG1** | OX40L | ICOS | p53 | INPP4B | FOXP3 | PR |
| **PD-1/ PD-L1** | STING | PD-L2 | PARP | PLCG1 |  | PTEN |
| **Rb IgG/ GAPDH** | TIM-3 |  | Cleaved Caspase9 | Phospho-PRAS40 (T246) |  | S100B |
| **CD56** | VISTA |  | Neurofibromin |  |  |  |
| **GZMB** |  |  |  |  |  |  |
| **SMA/ Fibronectin** |  |  |  |  |  |  |
| **CD68** |  |  |  |  |  |  |
| **HLA-DR** |  |  |  |  |  |  |
| **CTLA4** |  |  |  |  |  |  |
| **PanCk/Ki-67** |  |  |  |  |  |  |

*Supplementary Table 2. List of antibodies in the panel..*

| **Target** | **Clone** | **Dilution** | **Vendor** |
| --- | --- | --- | --- |
| **CD4** | EPR6855 | 1:200 | Abcam |
| **CD68** | KP1 | 1:200 | ThermoFisher Scientific |
| **CD20** | L26 | 1:200 | ThermoFisher Scientific |
| **CD11c** | 118/A5 | 1:200 | ThermoFisher Scientific |
| **CD45RO** | UCHL1 | 1:50 | BioLegend |
| **HLA-DR** | EPR3692 | 1:50 | Abcam |
| **Ki67** | B56 | 1:200 | BD |
| **CD3e** | EP449E | 1:200 | Abcam |
| **PanCk** | AE-1/AE-3 | 1:200 | BioLegend |
| **CD34** | QBEND10 | 1:50 | Abcam |
| **CD31** | EP3095 | 1:200 | Abcam |
| **Podoplanin** | NC-08 | 1:200 | BioLegend |
| **CD8** | C8/144B | 1:200 | BioLegend |
| **CD21** | EP3093 | 1:200 | Abcam |
| **HLA-A** | EP1395Y | 1:50 | Abcam |
| **CD45** | D9M81 | 1:200 | Cell Signaling Technology |
